# Robot‐assisted radical nephrectomy and inferior vena cava tumor thrombectomy: Initial experience in Japan

**DOI:** 10.1002/iju5.12419

**Published:** 2022-01-25

**Authors:** Daisuke Motoyama, Toshiki Ito, Takayuki Sugiyama, Atsushi Otsuka, Hideaki Miyake

**Affiliations:** ^1^ 12793 Department of Urology Hamamatsu University School of Medicine Hamamatsu Japan

**Keywords:** inferior vena cava tumor thrombectomy, renal cell carcinoma, robot‐assisted radical nephrectomy

## Abstract

**Introduction:**

Open surgical approach remains the standard treatment for renal cell carcinoma with an inferior vena cava tumor thrombus. In recent years, however, robot‐assisted radical nephrectomy and inferior vena cava tumor thrombectomy have emerged as minimally invasive alternatives to conventional open surgery.

**Case presentation:**

Here, we describe a 76‐year‐old female patient with right renal cell carcinoma with a level I inferior vena cava thrombus undergoing robot‐assisted radical nephrectomy and inferior vena cava tumor thrombectomy, which was successfully completed with a purely robotic procedure, resulting in the following outcomes: console time,167 min; total operative time, 211 min; and estimated blood loss, 150 mL. To our knowledge, this is the first case managed by robot‐assisted radical nephrectomy and inferior vena cava tumor thrombectomy in Japan.

**Conclusion:**

Based on our experience, it might be worthwhile to consider purely robotic surgery for the treatment of renal cell carcinoma with an inferior vena cava thrombus.

**Abbreviations & Acronyms**
IVC: inferior vena cava
RA‐RN/IVCTT: robot‐assisted radical nephrectomy and inferior vena cava tumor thrombectomy
RCC: renal cell carcinoma
RN: radical nephrectomy

**Keynote message**

This is the initial report describing a patient undergoing RA‐RN/IVCTT in Japan, and our experience suggests that it is worthwhile to consider a purely robotic procedure as the surgical treatment for RCC with an IVC thrombus.

## Introduction

One of the most unique features of RCC is its tendency to extend to the IVC.^1^ Although RN and IVCTT by open surgery remain the standard management for such cases, recent advances in minimally invasive surgery have encouraged surgeons to perform this challenging procedure with a robotic approach.^2^ In fact, since the initial report by Abaza *et al*. in 2011,^3^ several studies have reported promising findings for RA‐RN/IVCTT.^2^, ^3^, ^4^, ^5^, ^6^, ^7^ However, this type of robotic surgery is currently performed solely by well‐experienced surgeons in limited high‐volume centers, and RARN has yet to be approved by the health insurance system; thus, RA‐RN/IVCTT has not yet been performed in Japan.

In this report, we describe the first experience of RA‐RN/IVCTT involving a patient with RCC and an IVC tumor thrombus corresponding to level I, which was successfully completed with a purely robotic procedure.

## Case presentation

**Fig. 1:**
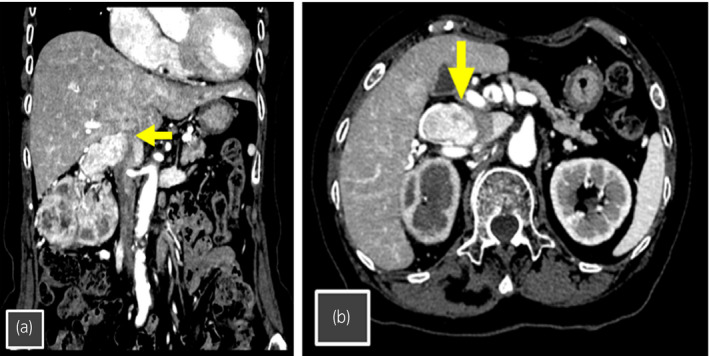
Computed tomography showing right RCC with a level 1 IVC tumor thrombus (arrow). (a) Coronal section. (b) Axial section.

A 76‐year‐old woman was referred to our department due to the diagnosis of a right renal mass. Radiological examinations showed an enhancing right renal mass (8.9 cm) and an IVC tumor thrombus corresponding to level I, without any findings suggesting metastatic diseases (Fig. 1). Considering her favorable general condition, RA‐RN/IVCTT was scheduled to be performed. As described previously, we have started RARN after the approval by the research ethics committee of our hospital (Certificate number: 21‐091),^8^ and written informed consent by this patient to receive RARN was obtained.

**Fig. 2:**
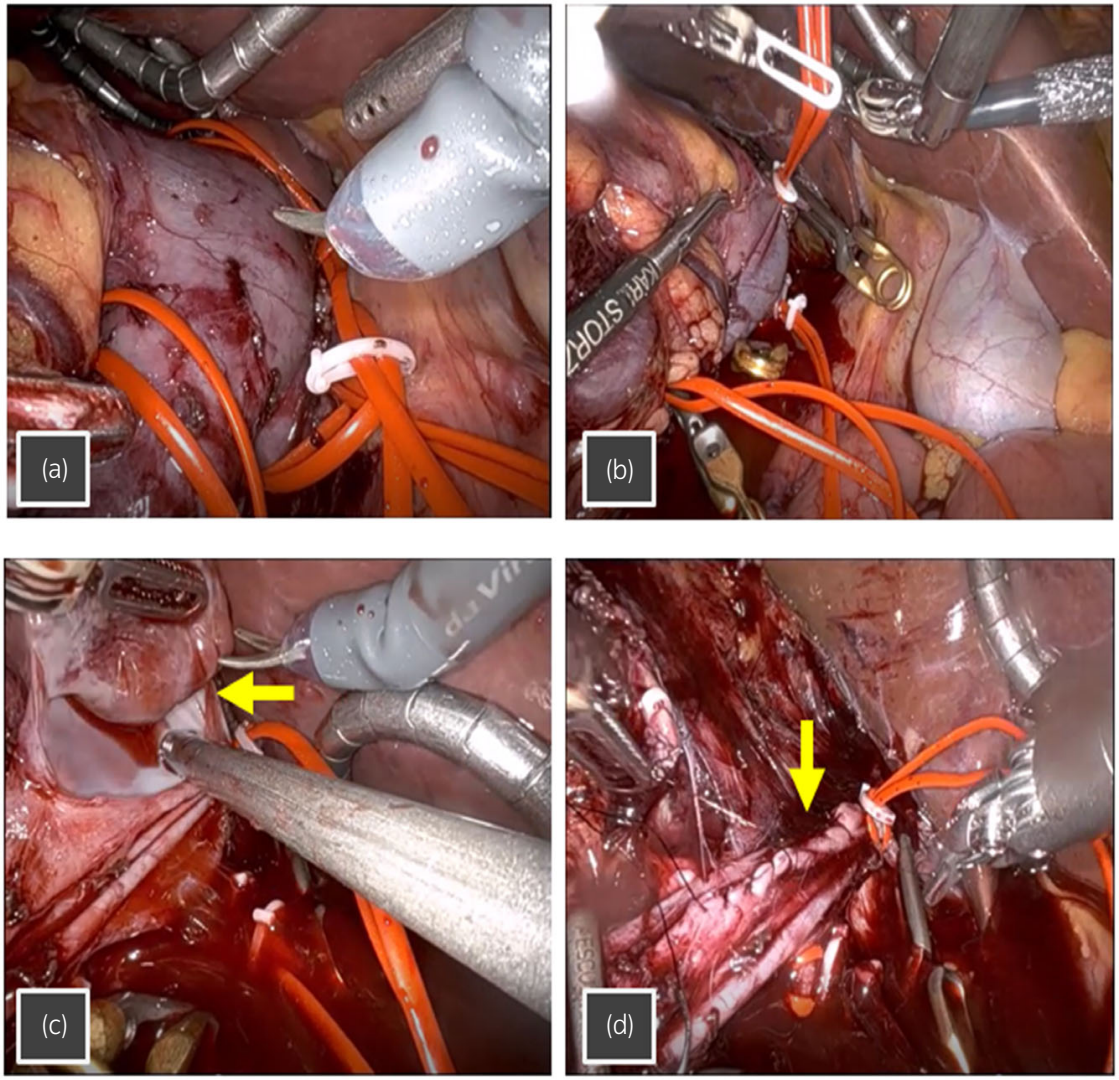
(a) The left renal vein, caudal IVC, and cephalic IVC secured by the twice‐wrapped vessel loops, and (b) sequentially clamped with the vessel loops closely by clipping in addition to the use of bulldogs. (c) The tumor thrombus (arrow) was removed from inside the IVC, after the wall of IVC was cut. (d) The IVC reconstructed with 4‐0 polypropylene suture (arrow), following the removal of the tumor thrombus.

The patient’s position and placement of trocars were same as those in our previous report.^8^ After the robotic system was docked, under the cephalic retraction of the liver, the right colon and duodenum were medially reflected, and the surfaces of bilateral renal veins and IVC were exposed. To circumferentially dissect IVC above and below the renal hilum, all feeding veins were divided after clipping. The left renal vein was dissected and secured by a twice‐wrapped vessel loop. In the inter‐aortocaval space, the right renal artery was exposed, double clipped, and transected. Subsequently, the location of tumor thrombus in the IVC was visualized with a laparoscopic ultrasound probe to confirm the upper limit of the IVC thrombus, and IVC was secured above and below the thrombus by twice‐wrapped vessel loops (Fig. 2). The left renal vein, caudal IVC, and cephalic IVC were clamped sequentially with the vessel loops closely by clipping in addition to the use of bulldogs. The IVC wall near the renal hilum was cut, the tumor thrombus was completely removed from the IVC, and caval reconstruction was done using 4‐0 polypropylene suture. The proximal end of IVC, the distal end of IVC, and the left renal vein were sequentially released to restore caval flow. After IVCTT was completed, right RN combined with an en bloc removal of the right adrenal gland was conducted.^8^

**Fig. 3:**
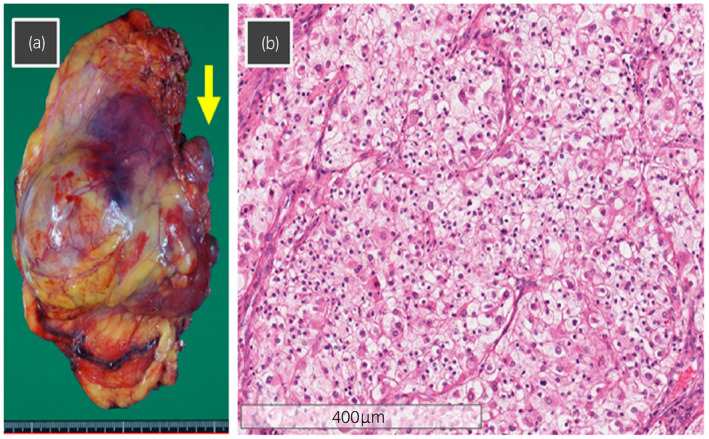
(a) Macroscopic findings of the excised right renal tumor and IVC tumor thrombus (arrow) with an en bloc removal of the right adrenal gland. The excised weight was 385 g. (b) Microscopic findings of hematoxylin and eosin staining showing clear cell RCC, pT3b and Fuhrman grade 4.

The console time, total operative time, and estimated blood loss were 167 min, 211 min, and 150 mL, respectively, and no significant complication was noted during or after RA‐RN/IVCTT. Five days after the operation, this patient was discharged. Pathological examination revealed the following findings: clear cell RCC, pT3b, and Fuhrman grade 4 (Fig. 3).

## Discussion

Since the first description,^3^ robotic surgery for RCC with a tumor thrombus in the IVC has been increasingly utilized as a minimally invasive alternative to a traditional open surgery, and outcomes of robotic surgery were shown to be feasible, leading to significantly small blood loss and a short hospital stay compared with open approach.^2^, ^3^, ^4^, ^5^, ^6^, ^7^ More recently, therefore, a robotic approach has been expanded to RCC with a high‐level IVC tumor thrombus by a few teams with sufficient experience of robotic surgery.^9^, ^10^ In Japan, RA‐RT/IVCTT has not been performed, because RARN has yet to be approved; however, we have conducted RARN after the approval by the research ethics committee.^8^ Based on this experience, we firstly applied a purely robotic approach to the treatment of a patient diagnosed with RCC with an IVC thrombus in Japan.

In this case, no significant complication occurred, resulting in the achievement of satisfactory perioperative outcomes. In addition, contrary to previous studies showing a longer operative time,^2^, ^3^, ^4^, ^5^, ^6^, ^7^ the robotic procedure in this case could be completed within 3 h. These favorable outcomes could be explained, at least in part, by the extensive experience of the operator, who has been involved in open surgery for >100 cases with RCC and an IVC thrombus as well as robotic renal surgery for >300 cases, including partial nephrectomy, RN, and pyeloplasty. Accordingly, if performed by a well‐experienced surgeon, purely robotic surgery could be a reasonable approach for the treatment of RCC with an IVC thrombus corresponding to level I.

Here, we would like to describe important issues associated with this case. First, in this case, IVC and the left renal vein were clamped by both the twice‐wrapped vessel loops and bulldogs in careful preparation for massive bleeding. However, only one item, mainly either the Rummel tourniquet or a modified technique, like that in this case, was reported to be used in previous studies^2^, ^3^, ^4^, ^5^, ^6^, ^7^; therefore, it should be considered to clamp them with the twice‐wrapped vessel loops alone to simplify the procedure. Second, when applying the robotic approach in the next case, it will be necessary to discuss whether omitted procedures in this case, such as irrigation of the caval lumen and covering of the removed thrombus with a specimen bag, should be introduced. Finally, expansion of the indication of the robotic approach to RCC with an IVC thrombus ≥level II will be expected; however, several additional procedures, such as control of the porta hepatis,^9^, ^10^ will be required to realize this.

In conclusion, this is the first report describing successful treatment with RA‐RN/IVCTT in Japan, and our experience suggests that it might be worthwhile to consider a purely robotic approach for the surgical treatment of RCC with an IVC thrombus.

## Author Contributions

Daisuke Motoyama: Conceptualization; Data curation; Investigation; Methodology; Validation; Writing – original draft. Toshiki Ito: Investigation; Methodology; Project administration. Takayuki Sugiyama: Supervision. Atsushi Otsuka: Supervision. Hideaki Miyake: Conceptualization; Methodology; Supervision; Validation; Writing – review & editing.

## Conflict of interest

The authors declare no conflict of interest.

## Approval of the research protocol by an Institutional Reviewer Board

21‐091.

## Informed consent

Not applicable.

## Registry and the Registration No. of the study/trial

Not applicable.
